# Helping While Social Distancing: Pathogen Avoidance Motives Influence People’s Helping Intentions during the COVID-19 Pandemic

**DOI:** 10.3390/ijerph182212113

**Published:** 2021-11-18

**Authors:** Yi Ding, Tingting Ji, Yongyu Guo

**Affiliations:** School of Psychology, Nanjing Normal University, Nanjing 210097, China; yiding2017@hotmail.com (Y.D.); yyguo@njnu.edu.cn (Y.G.)

**Keywords:** COVID-19, behavioral immune system, disgust sensitivity, helping, social distancing, anxiety

## Abstract

The behavioral immune system (BIS) theory suggests that pathogen avoidance motives relate to greater behavioral avoidance against social interactions that pose potential risks of pathogen transmission. Based on the BIS theory, pathogen avoidance motives would decrease people’s helping behavior towards others. However, would pathogen avoidance motives decrease all types of helping behavior towards others during the Coronavirus disease 2019 (i.e., COVID-19) pandemic indiscriminately? In the present study, we conducted a within-subjects design to compare people’s helping intentions toward voluntary work with and without social contact. Specifically, participants (*N* = 1562) completed an online survey at the early stage of the COVID-19 pandemic in China measuring pathogen disgust sensitivity, state anxiety, and intentions to perform volunteer work with and without social contact. Results revealed that pathogen disgust sensitivity negatively predicted intentions to perform voluntary work with social contact yet had no influence on intentions to perform socially distanced voluntary work. Moreover, the effect of pathogen disgust sensitivity on socially distanced volunteering preference was mediated by the state anxiety people experienced during the pandemic. The findings have implications for understanding people’s helping behavior during the pandemic.

## 1. Introduction

First identified in December 2019, the Coronavirus disease 2019 (i.e., COVID-19) soon spread throughout China and subsequently became a global health crisis. On 11 March 2020, the World Health Organization labeled the outbreak a pandemic [1]. The World Health Organization later announced a set of preventive measures, including frequent hand washing, wearing facial masks, and social distancing [2]. Due to COVID-19’s reputation of being highly contagious, social distancing was considered a crucial practice in limiting the spreading of the coronavirus, especially at the early stage of the pandemic before the vaccines were developed. Meanwhile, as the pandemic spread and the later lockdowns started, there was an increasing need for volunteers, for example, volunteers who support people affected by the pandemic, such as shopping and delivering food supplies to people quarantined at home, driving healthcare staff, raising funds and donations, and providing psychological and emotional support through helplines. Notably, some of this voluntary work poses relatively high risks of pathogen transmission via frequent interpersonal contact. In this respect, how do people navigate a trade-off in which helping others might expose them to the risk of contamination? Based on the behavioral immune system (BIS) framework, the present research aims to explain how individual differences in pathogen avoidance motives may influence people’s help intentions toward different kinds of voluntary work (i.e., with/without social contact) at the early stage of the COVID-19 outbreak in China.

Since the announcement of the preventative rules for stopping the spread of the coronavirus, there has been a growing number of studies to identify the influence of COVID-19 on people’s preventive behaviors, including social distancing [3,4]. A set of psychological mechanisms might be relevant for understanding such a process, particularly the BIS framework, which mainly focuses on explaining how people detect and avoid signs of infectious disease [5,6]. The BIS is thought to be an evolved mechanism in facing the adaptive challenge of pathogen threats that appeared throughout human evolutionary history [7,8]. In addition, compared to our physiological immune system, the BIS acts as the “first line of defense” against pathogens [9]. Specifically, the BIS motivates individuals to pay attention to cues of pathogens (e.g., coughs and sneezes), respond to sources of pathogens with specific emotions such as disgust and anxiety [7,10], and more importantly, generate avoidance-oriented behaviors and attitudes toward those displayed potential infectious symptoms [11,12,13]. For example, the BIS literature suggests that human pathogen avoidance adaptations motivate prejudice against morphologically anomalous individuals (e.g., people who are physically disabled, obese, or elderly [14,15,16]) and individuals of foreign origin (e.g., immigrants [17,18]).

According to the BIS theory, there are trade-offs of investing in pathogen avoidance behaviors [6,8]. It might be beneficial if people strictly avoid all direct and indirect contact with other people during COVID-19. At least avoidance minimizes the possibility of exposure to the coronavirus that is directly carried by other human beings. Consistent with this idea, a cross-cultural study has shown that people who live in regions with high levels of infectious disease often perform fewer normative physical contacts in domains of greetings, romantic kissing, and mortuary practice [19]. However, avoiding all social contact poses potential costs, such as eliminating chances of shopping for food and supplies, having sex, and taking care of families [20]. Consequently, a well-functioning BIS should balance the costs and benefits of behavioral avoidance of social contact [8]. That is, the cost–benefit ratio of any social contact avoidance is likely to vary across individuals and contexts.

At the individual differences level, people who have stronger pathogen avoidance motives (e.g., high on pathogen disgust sensitivity) might experience more severe emotional distress during COVID-19 outbreaks and are more likely to avoid social contact than others. Consistent with this possibility, previous studies found that individuals who have higher sensitivity toward pathogen cues reported greater anxiety disorders during the H1N1 “swine flu” [21,22], the Ebola outbreaks [23], and the COVID-19 pandemic [24]. In addition to emotional distress, they also reported increased engagement in social distancing during the pandemic [4]. As such, the present research predicts that individuals with higher pathogen avoidance motives would experience higher levels of emotional distress due to the COVID-19 pandemic and show more significant avoidance against social contact.

At the contextual level, researchers have uncovered that when the pathogen threat is made salient, people are more likely to avoid social contact with high-risk of infection, such as hugging and shaking hands with strangers [25]. Indeed, volunteering during the COVID-19 pandemic should be one of those high-risk of infection contexts that people may want to avoid. Help-workers and volunteers not only put themselves at risk of infection but also risk undermining their mental health [26]. For example, evidence suggests that volunteers and medical staff suffered more from insomnia, anxiety, depression, and distress [27]. However, not all voluntary work is equally likely to pose a threat of pathogen transmission. They vary in interpersonal physical distances: some are more frequently involved in face-to-face contact (e.g., hospital volunteering vs. volunteering with helplines) and, therefore, have higher risk of infection than others. Hence, we predicted that pathogen avoidance motives would only influence intentions to engage in volunteer work with social contact but have no influence on intentions to engage in socially distanced volunteer work.

In summary, based on the BIS framework, the present study predicted that people who have stronger pathogen avoidance motives (i.e., high on pathogen disgust sensitivity) would show less helping intentions toward voluntary work, especially voluntary work that includes potential social contact. In addition, since volunteers may experience more severe emotional distress during the pandemic, we predicted that anxiety experienced during the pandemic might mediate the influence of pathogen avoidance motives on helping intentions.

## 2. Materials and Methods

### 2.1. Participants and Design

Data from the present study was part of a large research project conducted in China from 31 January to 1 February 2020, one week after the lockdown of Wuhan City, Hubei Province, China. Ethical approval was obtained from the Ethics Committee of Nanjing Normal University (protocol code NJNU-2019-SYLL-021). A total of 1562 participants (719 females (46%), aged between 19 and 59, with a mean age of 31.31 years and SD of 8.14) were recruited online from Tencent Questionnaire (https://wj.qq.com, accessed on 31 January 2020), an online platform in China, which is similar to Amazon’s Mechanical Turk. Further, 36.2% of the participants were from Hubei Province.

Participants completed a consent form at the beginning of the survey and received a small amount of money in return after participation. To manipulate voluntary type, a one-way within-subject design was conducted (voluntary type: with social contact vs. socially distanced). After reading a short description of the COVID-19 pandemic situation in China, participants had to indicate their intentions toward both types of voluntary work.

### 2.2. Measures

#### 2.2.1. Helping Intentions

After reading a short description of the COVID-19 pandemic situation in China, participants rated their volunteer intentions toward two different types of voluntary work (i.e., with/without social contact). One of the types of voluntary work is collecting scientific materials and making flyers about coronavirus, which takes about two hours, and the working location is participants’ own apartments (i.e., the socially distanced voluntary work). The other volunteer work is distributing these flyers door to door to the neighbors. Similar to making flyers, flyer distributing also takes about two hours, and the location is participants’ neighborhoods (i.e., voluntary work with social contact). Helping intentions were measured on 7-point scales, from 1 (not at all) to 7 (very much), indicating how much they are willing to participate in the work.

#### 2.2.2. Pathogen Disgust Sensitivity (PDS)

To measure individual differences of pathogen avoidance motives, we used the pathogen disgust sensitivity subscale (Cronbach’s α = 0.81) from the Three Domain Disgust Scale (TDDS) [28]. PDS measures people’s sensitivity to pathogen-related disgust across seven items on 7-point scales (1 = Not at all disgusting, 7 = Extremely disgusting). Example items include “Stepping on dog poop” and “Shaking hands with a stranger who has sweaty palms.”

#### 2.2.3. State Anxiety

Participants completed the 6-item short-form of the state anxiety inventory (Cronbach’s α = 0.87) [29] on a 7-point scale (1 = strongly disagree, 7 = strongly agree), with items such as “I feel upset.” The higher scores indicate higher levels of anxiety.

#### 2.2.4. Demographics

In addition to the primary measures, participants also provided demographic information, including age, gender, educational background, and average personal monthly income. Table 1 presents the demographic characteristics of the participants. The sample in our study is likely representative of the general population in China.

### 2.3. Data Analyses

We first conducted a common method variance test to ensure no obvious common methodological bias in the present study. Next, correlations between variables (e.g., PDS, state anxiety, and voluntary intentions) were tested using Pearson’s correlation coefficient.

To test the effect of PDS and the within-subjects effect of voluntary type (with social contact vs. socially distanced) on people’s helping intentions, we conducted a mixed model. In the model, helping intentions were regressed on PDS, voluntary type, and their interaction. Next, although not a main focus of this study, we also added participants’ gender and age in an additional model to test the potential influences of gender and age on people’s helping intentions.

In addition, we conducted a mediation model to test the mediation role of state anxiety on the effect of PDS on socially distanced voluntary work preference. The delta value of helping intentions toward two types of voluntary work (i.e., socially distanced voluntary work preference) was the dependent variable, PDS was the independent variable, and state of anxiety was the mediator.

## 3. Results

### 3.1. Common Method Variance Test

To test the common method variance in this study, we conducted Harman’s Single Factor Test [30]. The test showed that the first factor accounted for 27.77% of the total variance and did not explain most of the variance (<40%). Thus, there was no obvious common methodological bias in this study.

### 3.2. Correlations

Table 2 shows the means and standard deviations of all primary variables and their correlations. As expected, PDS and state anxiety negatively and significantly correlated with helping intentions to voluntary work with social contact (*ps* < 0.001), but they did not significantly correlate with helping intentions to socially distanced voluntary work (*ps* > 0.35). In addition, PDS was positively and significantly correlated with state anxiety (*p* < 0.001).

### 3.3. Main Analyses

**Figure 1:**
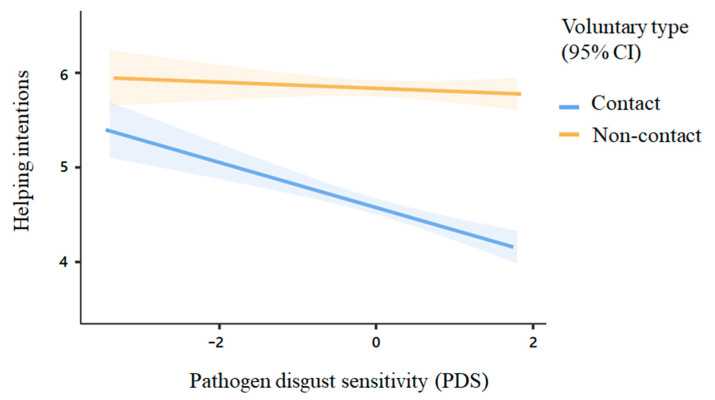
The effect of PDS on helping intentions toward different types of voluntary work. Note. Shadows represent 95% confidence intervals (i.e., 95% CI).

To directly test the main hypothesis that PDS negatively predicts people’s helping intentions to voluntary work involving potential social contact, we conducted a mixed model. In the model, PDS, voluntary type, and their interaction were used to predict helping intentions. Firstly, we found a significant main effect of PDS (estimate = −0.14, *t* = −3.77, *p* < 0.001), suggesting that individuals with higher PDS reported fewer helping intentions toward voluntary work. Next, we also found a significant main effect of helping type (estimate = 0.62, *t* = 25.57, *p* < 0.001), suggesting that people are more willing to participate in socially distanced voluntary work (*M* = 5.83, *SD* = 1.42) than work with social contact (*M* = 4.59, *SD* = 1.95). In addition, the interaction between PDS and voluntary type was also significant (estimate = 0.10, *t* = 4.25, *p* < 0.001). To further probe the interaction, we conducted a simple slope test (see Figure 1). We found that PDS only negatively predicted helping intentions toward voluntary work with social contact (estimate = −0.24, *p* < 0.001) but did not affect socially distanced voluntary work (estimate = −0.03, *p* = 0.484), which is consistent with our hypothesis.

Although not a main focus of the present study, we also tested the potential influences of gender and age on participants’ helping intentions. We did so by adding gender, age, and their interactions with PDS and voluntary type in an additional mixed model as an exploratory analysis. Effects of PDS and the interaction of PDS and voluntary type remained the same.

**Figure 2:**
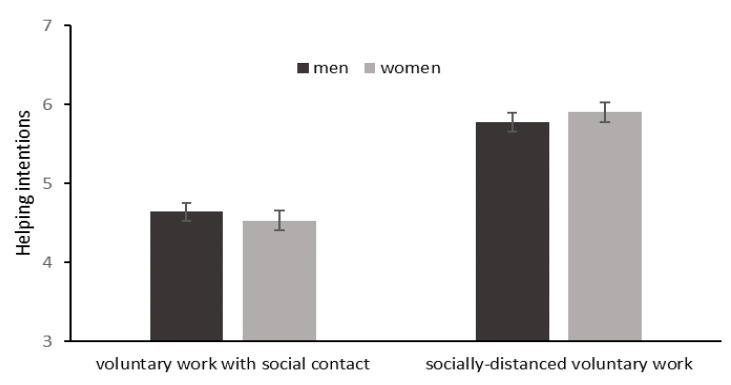
The effect of gender on helping intentions toward different types of voluntary work. Note. Error bars represent 95% confidence intervals.

Regarding gender effects, the results did not show a significant main effect of gender (estimate = 0.01, *t* = 0.18, *p* = 0.858). However, the interaction between gender and voluntary type was significant (estimate = 0.06, *t* = 2.43, *p* = 0.015), see Figure 2. Further, simple effect analysis found that women (*M* = 4.51, *SD* = 1.42) showed fewer—but not statistically significant—helping intentions toward voluntary work with social contact than men (*M* = 4.65, *SD* = 1.96; estimate = −0.05, *p* = 0.219). In contrast, women (*M* = 5.90, *SD* = 1.38) showed greater—but not statistically significant—helping intentions toward socially distanced voluntary work than men (*M* = 5.78, *SD* = 1.45; estimate = 0.07, *p* = 0.128).

**Figure 3:**
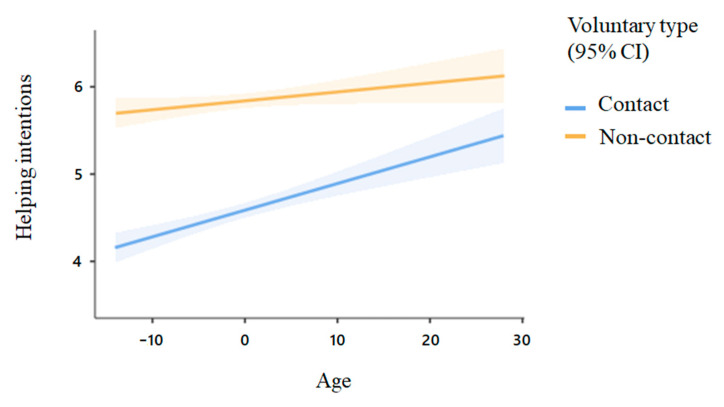
The effect of age on helping intentions toward different types of voluntary work. Note. Shadows represent 95% confidence intervals.

Regarding age effects, we found a significant main effect of age (estimate = 0.02, *t* = 4.54, *p* < 0.001), suggesting that older people showed greater helping intentions. In addition, there was a significant interaction between age and voluntary type (estimate = −0.01, *t* = −3.30, *p* = 0.001), see Figure 3. Results of a further simple slop test showed that age significantly predicted helping intentions toward voluntary work with social contact (estimate = 0.03, *p* < 0.001), while only marginally predicted helping intentions toward socially distanced voluntary work (estimate = 0.01, *p* = 0.061).

### 3.4. Mediation Analysis

**Figure 4:**
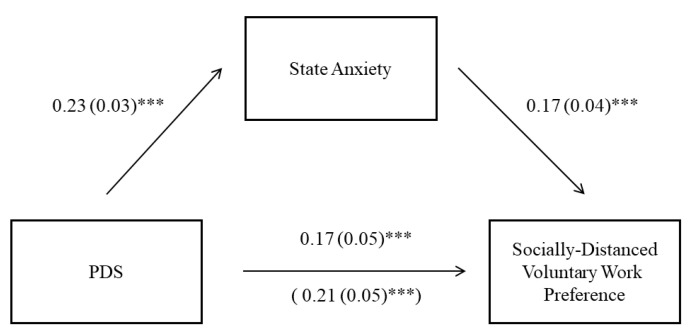
Mediation effect of state anxiety on the association between pathogen disgust sensitivity (PDS) and socially distanced voluntary work preference. Note. Significance of non-standardized regression coefficients (and standard error) is indicated. *** *p* < 0.001.

To further investigate whether state anxiety mediated the effect of PDS on preference for socially distanced voluntary work, we conducted a mediation analysis in which the delta value of helping intentions toward two types of voluntary work (Intention _non-contact_ − Intention _contact)_ was the dependent variable, PDS was the independent variable, and state anxiety was the mediator (see Figure 4). Results showed a significant indirect effect of state anxiety on the relationship between PDS and preference to participate in socially distanced voluntary work: estimate = 0.04, 95%CI (0.02, 0.06). This finding suggests that people with higher sensitivity toward pathogen-related disgust might have experienced higher levels of state anxiety during the pandemic and therefore showed greater avoidance against social contact while helping others.

## 4. Discussion

Worldwide, the COVID-19 pandemic has brought an unprecedented pathogen threat to people’s health and life. The BIS is suggested to be the pathogen avoidance mechanism that motivates people to inhibit contact with potential pathogen transmission in the first place. Based on the BIS theory, the main goal of the present study was to investigate how individual differences in pathogen avoidance motives (e.g., pathogen disgust sensitivity) may influence people’s helping intentions towards two distinct types of voluntary work (i.e., with and without social contact) during the COVID-19 pandemic. We found that PDS negatively predicted helping intentions toward voluntary work with potential social contact yet had no influence on helping intentions toward socially distanced voluntary work. In addition, we found a mediating role of state anxiety on the relationship between PDS and socially distanced voluntary work preference. Our findings are consistent with the BIS framework and suggest the implication of the BIS on understanding people’s helping behavior during the COVID-19 pandemic.

The findings showed that high sensitivity to pathogen-related disgust was associated with greater avoidance of voluntary work that involves potential social contact, which is in line with predictions derived from the BIS framework on human sociality. Growing evidence suggests that the BIS does not promote the same pathogen avoidance motivations toward all individuals and across all contexts indiscriminately. Instead, it is functionally flexible [31]. The BIS is sensitive to contextual information indicating an infection-risky environment and people who are chronically anxious and worry about pathogen infection [32,33]. The current findings suggest that participating in voluntary work that involves potential social contact during the pandemic might be one of those infection-risky contexts that the BIS motivates people to avoid. These findings are also consistent with recent work suggesting that PDS was associated with greater engagement in preventative health behaviors such as following social distancing rules [4].

Importantly, we found a mediating role of state anxiety on the association of PDS and socially distanced voluntary work preference, which suggested that people who are more sensitive to pathogen-related disgust might have experienced higher state anxiety levels during COVID-19. Such anxiety would further promote health-protective behavior (e.g., helping but social distancing) against social contact with high-risk of infection. This finding is consistent with evidence suggesting that PDS is associated with more severe stress and anxiety disorders [24,34], especially during disease outbreaks. For example, people with higher disgust proneness reported greater fear and anxiety during the 2009–2010 H1N1 “swine flu” [21,22], the 2014–2015 Ebola outbreaks [23], and the COVID-19 pandemic [35]. We should note that anxiety responses might be a double-edged sword during the pandemic. On the one hand, anxiety response facilitates people’s engagement in social distancing, which is crucial in limiting the spread of the coronavirus. On the other hand, anxiety response results in a significant decrease in people’s prosocial intentions. In fact, evidence shows that COVID-19 volunteers are practically vulnerable to mental distress such as depression, anxiety, and somatization [26,27,36]. Future studies could further explore the role of anxiety in volunteering during COVID-19.

A critical contribution of the present research is assisting the understanding of people’s helping intentions and behavior during the pandemic. Due to the growing pandemic, there is an increased need for volunteers. Normally, helping others (e.g., volunteering) has a positive influence on the helpers’ wellbeing, such as providing a sense of meaning and satisfaction [37]. However, COVID-19 makes some forms of volunteering particularly risky or impossible to undertake, especially amongst those who are sensitive to pathogen-related disgust. Based on the present findings, we suggest that more types of socially distanced volunteering should be offered during the pandemic, and individual differences in pathogen avoidance motives should be considered during volunteer recruitments.

Before closing, we will briefly outline the limitations and prospects for future research. First, theoretically speaking, using the BIS theory to explain psychological and behavioral responses against the pandemic in modern larger-scaled societies should be cautious [38]. The BIS evolved mainly in response to selection pressures of pathogen transmission in small-scale subsistence groups. Using the BIS to understand people’s helping behavior during COVID-19 requires a better understanding of when pathogen avoidance mechanisms work in the modern context. Next, in the present study, we tested the mediating role of anxiety experiences on the association of PDS and voluntary preference. However, we did not compare the anxiety levels of participating in different kinds of voluntary work. Do people actually experience higher levels of anxiety when facing social contact? Future studies could further test the mediating role of anxiety toward different voluntary work. In addition, our findings are based on a general population from China. We did not test the potential influences of some demographic characteristics, such as cultural background and religion. Although not a main focus of this study, future research could benefit from comparing cultural, religious, and occupational differences in the effect of pathogen avoidance motives on helping intentions during the COVID-19 pandemic. Finally, some recent research proposes that people’s comfort with social contact with high-risk of infection may depend on the interpersonal value of the target [39]. For example, people who valued their closest friend more were also more likely to contact and help that friend, even when there was a high disease risk. Future studies could benefit from comparing helping intentions during the pandemic toward targets with different interpersonal values.

## 5. Conclusions

Taken together, results from the present study suggest that people who have higher pathogen avoidance motives (i.e., pathogen disgust sensitivity) showed less willingness to participate in voluntary work that includes potential social contact. In contrast, pathogen disgust sensitivity did not influence helping intentions toward socially distanced voluntary work. In addition, we found a mediating role of state anxiety, indicating that people who have higher pathogen disgust sensitivity are more likely to experience anxiety distress during the pandemic, and therefore prefer to help others in a socially distanced way. As COVID-19 continues to spread and impact our lives worldwide, our findings could help increase understanding of people’s helping behavior during the pandemic.

## Figures and Tables

**Table 1:** Demographic characteristics of study participants (*N* = 1562).

Variable	Categories	Frequency	Percentage (%)
Age	19–24	299	19.1%
	25–30	592	37.9%
	31–40	443	28.4%
	41–50	175	11.2%
	51–60	53	3.4%
Gender	Women	719	46%
	Men	843	54%
Educational Background	Primary school or less	12	0.8%
	Middle school graduate	47	3%
	High school graduate or equivalent education completed	129	8.3%
	Junior college graduate	453	29%
	College graduate	757	48.5%
	Postgraduate degree	164	10.5%
Average Personal Monthly Income in Chinese Yuan (i.e., CNY)	CNY <1000	102	6.5%
	CNY 1000–2000	103	6.6%
	CNY 2000–3000	162	10.4%
	CNY 3000–5000	391	25%
	CNY 5000–8000	361	23.1%
	CNY 8000–12,000	251	16.1%
	CNY 12,000–15,000	82	5.2%
	CNY 15,000–20,000	42	3%
	CNY >20,000	63	4%

**Table 2:** Zero-order correlations among the primary variables.

Variable	M	SD	1	2	3	4
1. Pathogen disgust sensitivity	4.45	0.99	—			
2. State anxiety	5.00	1.28	0.18 ***	—		
3. Voluntary work (with social contact)	4.59	1.95	−0.12 ***	−0.14 ***	—	
4. Voluntary work (socially distanced)	5.83	1.42	−0.02	−0.02	0.38 ***	—

Note. *N* = 1562. *** *p* < 0.001.

## Data Availability

Data will be provided if requested of the authors.
